# Gendered Perceptions of Migration Among Ghanaian Children in Transnational Care

**DOI:** 10.1007/s12187-016-9407-x

**Published:** 2016-07-12

**Authors:** Victor Cebotari, Valentina Mazzucato, Melissa Siegel

**Affiliations:** 1 Maastricht Graduate School of Governance, Maastricht University, Boschstraat 24, 6211 AX Maastricht, The Netherlands; 2 Department of Technology and Society Studies, Maastricht University, Grote Gracht 82, 6211SZ Maastricht, The Netherlands

**Keywords:** Child agency, Migration, Children left-behind, Transnational families, Child gender, Africa, Ghana

## Abstract

This study empirically measures the perceptions towards maternal and paternal migration of male and female children who stay behind in Ghana. It analyses survey data collected in 2010 among secondary school children aged 11–18 in four urban areas with high out-migration rates: the greater Accra region, Kumasi, Sunyani and Cape Coast (*N* = 1965). The results show significant gendered differences in how children perceive parental migration. Specifically, female children have more positive views towards maternal and paternal migration when parents are abroad and in a stable marital relationship, when the assessed parent is abroad but the other parent is the caregiver in Ghana, when there is a frequent change in the care arrangement, and when female children receive remittances. These findings were not replicated for male children. The analysis highlights the sensitivity of the results to the gender of the child and to the characteristics of children’s transnational lives that are being analysed.

## Introduction

Living a transnational family life, in which at least one member of the nuclear family has migrated abroad, is a common feature among children in Ghana. It was estimated that by 2010, approximately 825,000 Ghanaians, excluding undocumented migrants, had migrated to search for better opportunities abroad (World Bank 2011). Other estimates put the number of Ghanaians migrants at 1.5 million, including those who migrated within Africa (chiefly to ECOWAS countries) and overseas to Europe, North America, Middle East and Asia (Twum-Baah 2005). Many of these migrants are parents who, either voluntarily or forced by the perils of the migration process, choose to leave their children behind in the care of the other parent, a relative or other caregivers, thus creating a network of transnational families. Although quantifying the exact number of children in transnational care is difficult, some statistics based on nationally representative data show that 37 % of Ghanaian children aged 0–18, excluding orphans, had one or both biological parents away in 2014 (GDHS 2014).

Despite the high proportion of children who live separated from their parents due to migration, relatively little is known about this population. The functioning of families across borders and the effects this has on individual family members have been the focus of transnational family studies for the past two decades (Baldassar and Baldock 1999; Bryceson and Vuorela 2002). Studies have focused primarily on the well-being outcomes of migrant parents and children in transnational care (Dreby 2010; Jordan and Graham 2012; Parreñas 2005) and to a lesser extent on the emotional experiences of being separated from loved ones in the context of migration (Milton and Svašek 2005; NíLaoire et al. 2010). As Baldassar (2008) notes, living transnationally, especially the perception of separation from kin, is a topic full of emotion. It is now increasingly accepted that children are autonomous social actors (Asis 2006; Dreby 2010) and that their experiences should be integrated into studies of the left behind because this context is also a result of their own actions and thus must be understood from their own perspectives (Hoang and Yeoh 2015).

This study aims to contribute to the literature of transnational families by empirically measuring the perceptions towards maternal and paternal migration of male and female children who live in transnational care in Ghana. Specifically, the contribution of this study is threefold. First, this study brings the complexity of children’s transnational lives into focus by investigating perceptions of migration by children who experience different transnational care arrangements. Within this setting, we distinguish between the marital status of the migrant parents, who the migrant parent is and who the caregiver is, the stability of the care arrangement and the availability of remittances. We distinguish between different characteristics to account for the diversity of the transnational child raising arrangements in Ghana.

Second, we employ a gendered perspective by analysing male and female children separately, and we compare the perceptions of children in transnational care with those in non-migrant families. Recent studies emphasize the need to engage appropriate control groups and to distinguish between the child’s gender when measuring children’s emotions (Mazzucato and Cebotari 2016; Vanore 2015).

Third, this study is the first large-scale empirical analysis of the relationship between parental migration and the experiences of children who stay behind in an African context. The current scholarship employs evidence predominately from Asia and Latin America and has a scant inclusion of the African transnational cases. The African geographical focus is important because many African countries have family norms that differ from the nuclear family model that guides much of the transnational family research in other parts of the world (Mazzucato et al. 2015a). In Ghana, as is the case in most Sub-Saharan African countries, the widespread practice of social parenthood, where children are raised by persons other than their biological parents, and child fostering, where children are entrusted to others to be cared for, may lead to different perceptions towards migration than those found in other geographical contexts.

This study uses data derived from a large-scale survey that was conducted in 2010 in Ghana among secondary school children aged 11–18. Perceptions of children towards maternal and paternal migration are analysed using logistic regression methods.

## Background

### Child Agency and Parental Migration

The topic of child agency and the context of parental migration has only recently captured the attention of scholars in transnational family research. In this study, the concept of agency is defined as socially unfettered free will that stimulates own perceptions based on rationality and self-awareness (Ahearn 2001). Most existing studies on children in transnational care have focused on the consequences of parental migration, treating children as passive actors, thus excluding their voices and experiences of living transnationally. The scholarship relies on paradigms stemming from childhood and family studies that perceive children as innocent human becoming that lack rationality and wisdom and have their needs met rather than their voices listened (Devine 2002; James et al. 1998). Recent evidence suggests that contemporary children are able to engage and act upon their experiences of living with migration (Hoang and Yeoh 2015).

Indeed, an emerging paradigm in transnational family research draws on sociological reflections of childhood that perceive children as autonomous social actors with their own perceptions of living in the realm of their parents’ departure following migration (Asis 2006; Dreby 2010; Hoang and Yeoh 2015; NíLaoire et al. 2010; Olwig 1999; Orellana et al. 2001; Parreñas 2005). Proponents of this paradigm conceptualize children in transnational care as independent beings rather than as innocent and dependent members of the family who need guidance and protection. An increasing number of scholars promote the idea that the lives of children in transnational care are shaped and transformed by their own actions; therefore, their perspectives towards migration must be integrated in studies of the left behind (Dreby 2010; Hoang and Yeoh 2015).

Research into children’s perspectives of migration shows that they are active actors in the decisions that concern their lives and those of the family members who migrate. In the Philippines, Asis (2006) reports that children perceive parental migration as an opportunity to escape from parental control to learn to take responsibility, resolve problems and face new opportunities in life. A study by NíLaoire et al. (2010) reveals that children are especially eager to negotiate and construct their own realms of migration, particularly when they plan to migrate themselves. Even in the face of family members’ migration when adults appear to be the decision-makers, children were found to actually influence the nature and course of their family members’ migration (Orellana et al. 2001). However, the extant research largely focuses on children and families with migration experience and less on the experiences of children in non-migrant families. Proponents of the new mobility paradigm (Sheller and Urry 2006) explored children’s conceptions of migration and concluded that both sedentary and mobile accounts of migration matter. In Mexico, Dreby (2007) found that children in non-migrant families tend to have limited influence over their parents’ initial migration decisions but that they were able to influence their parents’ subsequent migration trajectories.

It is also important to look at child agency and the migration history of children, as the experience of living transnationally is neither static nor universally uniform. In today’s globalised world, where it is relatively easy to travel between different locations, children of migrants were found to engage in frequent moves and for different reasons (Bledsoe and Sow 2011). Research by Wu et al. (2015) shows that children with a previous migration experience are more likely to exhibit signs of depression but not when they currently live with returned migrant parents. It implies, as other studies show, that child behaviour may play a role in parental (re)location decisions (Dustmann 2003). Moreover, migration studies emphasize that child agency vary with age and that different cultures treat children of a certain age differently. For instance, people in Ghana and in other West African countries may see teen children as active economic actors that are ready to generate income and support their families (Whitehead et al. 2007). In other contexts however, such as Vietnam, adults were found to perceive education as central for child development and to stigmatize the participation of children in paid work (Ennew et al. 2005). The positioning of children in different cultural contexts may relate directly to the way they exhibit views toward parental migration.

While there is agreement that children are role-players in the process surrounding their lives in transnational care, one must also recognize the limits of their perception of space and action (Ansell 2009). Scholars used terms such as ‘thin agency’ to describe the ambiguity in children’s perceptions of the world processes (Klocker 2007). This line of research builds on the argument that children do not always have the experience and the access to information as adults do and this limits their ability to act with a similar degree of awareness and intent. Studies also emphasize the need to recognize the importance of complex social and cultural norms that shape children’s lives in relation to migration (Cebotari and Mazzucato 2016; Mazzucato et al. 2015a). It is therefore important to situate the perceptions of children within the complexity of forms that transnational care involves and to confine research to the local contexts in which children reside.

### Children and the Complexity of Transnational Care Arrangements

It is now increasingly recognized that children of migrant parents live in different care arrangements that are variously gendered and contingent on context (Carling et al. 2012; Mazzucato and Cebotari 2016; NíLaoire et al. 2010). The literature on the Global South reveals intriguing accounts of children’s experiences following parental migration. In the context of the Philippines, Parreñas (2005) shows how the conventional gender division of labour between the mother and the father influences the way children experience parental migration. Specifically, children were found to readily accept the departure of their fathers who had migrated to fulfil the role of the breadwinner in the family. In contrast, children of migrant mothers often felt abandoned and tended to blame their mothers for their absence (Parreñas 2005). In addition, maternal migration was found to negatively affect the happiness status of children in a number of Southeast Asian countries (Jordan and Graham 2012). However, it is important to note that these studies looked solely at parental migration without considering the presence of the child’s caregiver who remained in the origin country. When accounting for who the caregiver is when parents migrate, recent studies found that children who stay in the care of a parent or another family member in Ghana and Moldova show no differences in emotional well-being compared with children in non-migrant families (Mazzucato and Cebotari 2016; Vanore 2015).

The role of the caregiver in children’s lives is therefore conceptually important but largely understudied in transnational family literature. Caregivers can influence how children perceive parental migration by speaking frequently to the children about their parents (Dreby 2007). In Sub-Saharan Africa child fostering is common, setting a culturally accepted norm of children being cared by persons other than their biological parents. Despite the role caregivers play in rearing children, little is known how stable the care arrangements are with regards to the frequency with which children of migrants change caregivers. To our knowledge, only two recent studies look at this dimension. In African contexts, Mazzucato and Cebotari (2016) and Mazzucato et al. (2015a) found that a frequent change in caregivers had negative consequences for the emotional well-being of children in transnational care. These findings warrant an investigation of whether caregiver stability has wider implications for children beyond their emotional distress.

Children’s perspectives have been little investigated when transnational families are affected by marital problems. When parents migrate, the stress of living apart can lead to marital tensions (Pribilsky 2004), especially when women migrate (Caarls and Mazzucato 2015). Research also reveals that migration can serve as an escape path from a problematic marriage for some Filipina wives (Constable 2003). In one of the few studies that looked explicitly at the transnational lives of children whose parents were either together or divorced, Mazzucato and Cebotari (2016) found that Ghanaian children were more emotionally distressed when their parents had migrated and were divorced than they were when their parents had migrated but remained in a stable marital relationship. In Mexico, research by Nobles (2011) shows that children experience parental migration and parental divorce in distinct ways and that parental divorce has a worse impact on children. These studies importantly suggest that children are likely to perceive migration differently when their parents are together versus when they are divorced. Migrant parents who are separated may not have the time and the necessary resources to invest in children, especially when they have formed new families abroad and have children in these new relationships (Dreby 2010).

Money through remittances is a core theme in the study of children who live in transnational families. The literature hints at the way children’s perceptions are influenced by the remitted capital. Many children of migrants perceive the destination countries as lands of abundant possessions, and migration is viewed as a source of gifts and money (Hoang and Yeoh 2015). In children’s eyes, remittances are not just money; they also represent the social presence of migrant parents and are often accepted by the children as a currency of love and care (Olwig 1999). Research in the Philippines also found that remittances are a successful strategy for maintaining warm relationships between migrant parents and their children (Parreñas 2005). These studies suggest that remittances can be a channel for children to construct their perceptions of migration despite the hardship of separation across national borders.

The literature also highlights the complex nature of children’s experience with migration, showing that it is also gendered. In the Philippines, it was shown that migrant mothers put more responsibility on their female children than on their male children by entrusting them with the care of younger siblings, remittances and with the overall household expenditure (Parreñas 2005). At the same time, boys who stay behind in Moldova were found to be more emotionally affected by parental migration, especially when their fathers had migrated and they were left in the care of a non-parental caregiver (Vanore 2015). In some African countries, the sharp separation of gender responsibilities was identified as one of the key characteristics of family life (Mazzucato et al. 2015b). In general, boys in Ghana were found to secure better work opportunities of higher monetary value, which provides them with both the incentive and the opportunity to migrate, but they are also easily persuaded to stay or to return (Whitehead et al. 2007). Ghanaian girls, in contrast, do primarily domestic work and need to be more resourceful in pursuing alternatives beyond their household duties. This context is believed to make Ghanaian girls perceive migration more positively because it provides them with a way to pursue better work opportunities abroad (Whitehead et al. 2007).

### Hypotheses

Taken together, the literature provides informative accounts of the contexts surrounding the transnational life of children. Importantly, it suggests that parental migration is not always associated with negative consequences for children and that factors such as the child’s gender and characteristics of the migrant and caregiving all have the potential to affect the way children perceive maternal and paternal migration. Based on the above-mentioned studies, the following hypotheses are explored.

- *Hypothesis 1a*: Parental migration and a stable marital relationship will more positively influence the perceptions towards parental migration of children in transnational care compared with those of children in non-migrant families.
- *Hypothesis 1b*: Parental migration accompanied by divorce will more negatively influence the perceptions towards parental migration of children in transnational care compared with those of children in non-migrant families.
- Hypothesis 2a: Children with a migrant mother and a caregiver father will be more likely than children in non-migrant families to perceive parental migration more negatively.
- *Hypothesis 2b*: Children with a migrant father and a caregiver mother will be more likely than children in non-migrant families to perceive parental migration more positively.
- *Hypothesis 2c*: Children with both parents abroad will be more likely than children in non-migrant families to perceive parental migration more negatively.
- *Hypothesis 3*: Children who frequently change caregivers will be more likely than children in non-migrant families who never changed caregivers to perceive parental migration more negatively.
- *Hypothesis 4*: The presence of remittances will positively influence how children perceive parental migration, whereas the absence of remittances is expected to negatively influence children’s perceptions towards parental migration compared with the perceptions of children in non-migrant families.

## Method

### Data

The data come from a survey that was conducted in 2010 among junior and senior secondary school children in Ghana. The survey took place in four urban areas with high out-migration rates: the greater Accra region, Kumasi, Sunyani and Cape Coast. The data are not representative at the national level, but detailed protocols were documented to allow for future replication.

Stratified sampling was applied to randomly select children from low- and high-quality and private and public schools in each urban area. The classification of schools was obtained from the Department of Education in Ghana, which divides public and private schools in different quality categories following exam scores and student enrolment rates. The sampling procedure resulted in the selection of 22 schools, and each was asked to participate. All accepted except for one school that refused and was replaced by another randomly selected school in the same urban area. In the schools, one class was randomly selected from each of the three grades. To ensure that a sufficient number of children in transnational families would be present in the data, we randomly selected an additional six classrooms from the three grades and pooled all children in transnational care, who then were asked to participate in the survey. We employed independent sample t-tests to compare the means of key characteristics in the pooled and randomly selected samples and found no significant variations. We therefore concluded that the inclusion of both samples did not affect the reliability of the data.

A questionnaire was designed to specifically capture the complexity of the lives of children in transnational care. The questionnaire was administered as a self-reporting tool that was completed by the students themselves under the guidance of the fieldwork team. In each classroom, the survey team was composed of a supervisor and up to three trained interviewers. Before administering the questionnaire, the team informed all students of the voluntary nature of the exercise, and the survey had an overall response rate of 85 %. The project and the survey were approved by the ethical committee of the University College Cork.

Overall, the data collected information from 2760 children. In this study, the students’ ages range between 11 and 18. To avoid ambiguity in the analysis, we chose to omit from the initial sample those pupils who were older than 18 (*N* = 266), those with at least one parent deceased, conditioned that no parent has migrated (*N* = 183) and those who did not know their parents’ locations at the time of the survey (*N* = 50). To isolate the role of international migration as the sole form of migration, we also omitted those children whose parents were away for work in different locations within Ghana (*N* = 296). Bivariate means comparison tests did not find significant differences between the excluded and non-excluded samples, attesting for a random exclusion of the observations.

### Measures

This study employs two dependent variables: one measuring the children’s perceptions towards maternal migration and the other measuring their perceptions towards paternal migration. Children were asked the following question: “In general, is it good or bad when fathers/mothers go away to work in another country?” Answers were based on a 5-point Likert scale (*very good, good, neither good nor bad, bad, very bad*). For analysis, binary versions were created where 1 indicated *very good* and *good*, and all other responses were coded as 0. The two measures were standardized as binary to simplify the comparison between the two independent variables and to better capture the positive variations in the outcomes. The rationale for dichotomization was documented and validated by previous research that used children’s reports to analyse a range of well-being outcomes (Jordan and Graham 2012).

Four major predictor variables were employed in this analysis relating to specific forms of life of children in transnational care. Looking at different transnational child raising arrangements is critical for understanding the complexity of migration and how this complexity is associated with children’s perceptions towards parental absence. The first variable measures the *location of the migrant parent and the type of separation* and includes categories for children in non-migrant families, children with parents away internationally and in a stable marital relationship and children with parents away internationally who are divorced or separated. The second indicator distinguishes between the *parental migration status and the caregiver*. This indicator has four categories: children in non-migrant families, children with the father abroad who are cared for by the mother, children with the mother abroad who are cared for by the father and children with both parents abroad and cared for by a non-parental caregiver. A third measure accounts for the *stability of the care arrangement* and has four categories: children in non-migrant families who never changed caregivers, children in non-migrant families who changed caregivers at least once, children with parents abroad who never changed caregivers and children with parents abroad who changed caregivers at least once. Finally, the fourth variable looks at the availability of *remittances* and has three categories: children in non-migrant families, children with parents abroad who receive remittances and children with parents abroad who do not receive remittances.

Additional indicators were included to control for child, family, and school characteristics. Child indicators included *age* and self-rated *happiness status* on a ten-point Likert scale to account for mental state and for respondents’ potentially inflated perceptions toward migration (Vanore 2015). Family indicators included binary variables for *maternal and paternal education*, with 1 indicating that the parent had completed secondary education or more; a continuous indicator that recorded the *total number of siblings*, including biological siblings, half-brothers and sisters, who lived with the respondent at the time of the survey; a binary variable that measured the *presence of younger siblings* living with the child; a self-declared binary indicator that assessed the *living conditions* of the child compared with other children, where 1 indicates better living conditions; a continuous indicator of *housing conditions*, where the total number of people living in the house was divided by the total number of rooms; and a binary family process indicator (Jordan and Graham 2012) that measured the *quality of the child-caregiver relationship*, where 1 denoted a distant relationship. School characteristics do not feature prominently in research pertaining to children in transnational care. In Ghana, there are large differences in the quality of education available to children (Cebotari and Mazzucato 2016). To control for variation in the school quality, we included two controls: one looking at *public* versus *private* schools and another outlining *low- or high-quality* schools based on official school ranking records in Ghana. Descriptive statistics of dependent and independent variables are presented in Tables 1 and 2.

**Table 1:** Means/percentages of dependent and independent variables

Variables	Full sample	
	% / mean (SD)	N/n
Dependent variable		
Children’s perception towards paternal migration is good/very good	36.84	724
Children’s perception towards maternal migration is good/very good	27.16	534
Independent variables		
Location parent(s) and the type of separation	100	1621
Non-migrant parents	67.86	1100
Parent(s) away internationally: together	23.01	373
Parents(s) away internationally: divorced/separated	9.13	148
Migration status and the caregiver	100	1411
Non-migrant parents	73.85	1042
Father away internationally, mother caregiver	14.53	205
Mother away internationally, father caregiver	2.76	39
Both parents away internationally, other caregiver	8.86	125
Stability of the care arrangement	100	1316
Non-migrant parents: changed never	57.45	756
Non-migrant parents: changed caregiver ≥1	16.03	211
Parent(s) away internationally: never changed caregiver	14.06	185
Parent(s) away internationally: changed caregiver ≥1	12.46	164
Remittances	100	1287
Non-migrant parents: no remittances	82.91	1067
Parent(s) away internationally: yes remittances	14.22	183
Parent(s) away internationally: no remittances	2.87	37
Child age (years)	15.33 (1.73)	1965
Child’s happiness status	4.16 (0.98)	1965
Mother’s education secondary or more	50.35	989
Father’s education secondary or more	72.82	1431
Nr. of siblings living with the child	3.01 (2.36)	1965
The child is living with younger siblings	55.15	1084
Living conditions are better when compared to other children	59.13	1162
Number of people per rooms in the house	1.56 (2.22)	1965
Distant relationship with the caregiver	23.68	465
Low–quality schools	32.64	641
Private schools	38.53	757

Standard deviations in parentheses. The n indicates the number of observations for categories within each indicator

**Table 2:** Perceptions towards paternal and maternal migration by child gender and transnational family characteristics

	Paternal migration is good/very good				Maternal migration is good/very good				
	Male		Female		Male		Female		
	%	Test statistic	%	Test statistic	%	Test statistic	%	Test statistic	N
Transnational family characteristics									
Location parent(s) and the type of separation		1.13		6.23^*^		2.11		3.09	1621
Non-migrant parents	41.07		31.44		28.25		22.14		1100
Parent(s) away internationally: together	42.33		41.76		34.38		28.57		373
Parents(s) away internationally: divorced/separated	34.48		34.72		30		25.71		148
Migration status and the caregiver		1.23		8.59^*^		7.29		9.32^*^	1411
Non-migrant parents	40.89		30.45		28.10		21.55		1042
Father away internationally, mother caregiver	39.36		41.05		28.57		22.83		205
Mother away internationally, father caregiver	42.86		42.11		35.71		42.11		39
Both parents away internationally, other caregiver	48.15		45.28		45.45		35.85		125
Stability of the care arrangement		0.53		8.44^*^		2.75		8.60^*^	1316
Non-migrant parents: changed never	41.74		29.12		30		19.95		756
Non-migrant parents: changed caregiver ≥1	41.18		35.24		27.38		28.85		211
Parent(s) away internationally: never changed caregiver	45.68		41.38		37.80		25.88		185
Parent(s) away internationally: changed caregiver ≥1	40.58		42.11		34.33		33.33		164
Receiving remittances		1.09		5.44		0.79		6.84^*^	1287
Non-migrant parents: no remittances	41.24		31.08		28.47		21.88		1067
Parent(s) away internationally: yes remittances	38.46		42.22		31.17		31.46		183
Parent(s) away internationally: no remittances	53.85		44.44		38.46		41.18		37

Chi-squared tests were used for all comparisons

* *p* < 0.05, ** *p* < 0.01, *** *p* < 0.001

### Analysis

The analysis used binary logistic regressions, and the results report the odds ratios with confidence intervals and the log-likelihood values for model specification. The analysis is conducted in two stages. Models in each stage are split by gender to accommodate eventual differences in reporting perceptions between male and female children. First, we examined the relationship between children’s perceptions and the status of being in a transnational family where at least one parent is abroad and where parents are either in a stable marital relationship or are divorced or separated. This stage is based on hierarchical regression modelling, in which we control in a stepwise fashion for child characteristics (Table 3, Models 1a & 1b; Table 4, Models 4a & 4b), family factors (Table 3, Models 2a & 2b; Table 4, Models 5a & 5b) and school type indicators (Table 3, Models 3a & 3b; Table 4, Models 6a & 6b).

**Table 3:** Stepwise binary logistic regressions predicting children’s perceptions towards *paternal* migration in relation to the location of parents and the type of separation

	Male						Female					
	Model 1a		Model 2a		Model 3a		Model 1b		Model 2b		Model 3b	
	*OR*	*CI*	*OR*	*CI*	*OR*	*CI*	*OR*	*CI*	*OR*	*CI*	*OR*	*CI*
Location parent(s) and the type of separation												
Non-migrant parents												
Parent(s) away internationally: together	1.13	[0.77,1.65]	1.03	[0.64,1.67]	1.10	[0.67,1.78]	1.71**	[1.18,2.48]	2.20**	[1.34,3.61]	2.33***	[1.41,3.85]
Parents(s) away internationally: divorced/separated	0.83	[0.46,1.52]	0.63	[0.26,1.55]	0.64	[0.26,1.57]	1.40	[0.82,2.39]	1.52	[0.76,3.06]	1.49	[0.74,2.96]
*X* ^2^	12.08		15.55		21.53		15.59		28.56		32.09	
Log–likelihood	−421.84		−272.83		−269.84		−466.56		−293.42		−291.66	

95 % confidence intervals (CI) in brackets; *OR* odds ratios; Male: *N* = 799; Female: *N* = 822

Models 1a & 1b include the age and the happiness status of the child.

Models 2a & 2b add the family characteristics (parents’ education; the number of siblings; younger siblings; living conditions; housing conditions; and the quality of child-caregiver relationship)

Models 3a & 3b add school type indicators (quality of schools; public-private schools)

* *p* < 0.05, ** *p* < 0.01, *** *p* < 0.001

**Table 4:** Stepwise binary logistic regressions predicting children’s perceptions towards *maternal* migration in relation to the location of parents and the type of separation

	Male						Female					
	Model 4a		Model 5a		Model 6a		Model 4b		Model 5b		Model 6b	
	*OR*	*CI*	*OR*	*CI*	*OR*	*CI*	*OR*	*CI*	*OR*	*CI*	*OR*	*CI*
Location parent(s) and the type of separation												
Non-migrant parents	–	–	–	–	–	–	–	–	–	–	–	–
Parent(s) away internationally: together	1.38	[0.92,2.08]	1.21	[0.72,2.03]	1.28	[0.76,2.17]	1.49	[0.98,2.27]	1.98*	[1.15,3.43]	2.01*	[1.16,3.49]
Parents(s) away internationally: divorced/separated	1.25	[0.67,2.33]	0.95	[0.37,2.43]	0.96	[0.37,2.48]	1.45	[0.79,2.63]	1.56	[0.73,3.32]	1.55	[0.73,3.31]
*X* ^2^	17.62		16.40		20.33		11.26		26.02		26.24	
Log–likelihood	−373.80		−241.38		−239.41		−392.31		−249.41		−249.30	

95 % confidence intervals (CI) in brackets; *OR* odds ratios; Male: *N* = 799; Female: *N* = 822

Models 4a & 4b include the age and the happiness status of the child

Models 5a & 5b add the family characteristics (parents’ education; the number of siblings; younger siblings; living conditions; housing conditions; and the quality of child-caregiver relationship)

Models 6a & 6b add school type indicators (quality of schools; public-private schools)

* *p* < 0.05, ** *p* < 0.01, *** *p* < 0.001

The second stage of analysis comprised three transnational characteristics—the migration status and the caregiver, the stability of the care arrangement and the availability of remittances — to assess how different transnational characteristics relate with children’s perceptions towards paternal (Table 5) and maternal (Table 6) migration. To isolate the sole effects of migration from those of marital discord, this stage of analysis omits all children whose parents were abroad and divorced or separated (*N* = 148).

**Table 5:** Children’s perceptions towards *paternal* migration in relation to different transnational family characteristics

	Male						Female					
	Model 7a		Model 8a		Model 9a		Model 7b		Model 8b		Model 9b	
	*OR*	*CI*	*OR*	*CI*	*OR*	*CI*	*OR*	*CI*	*OR*	*CI*	*OR*	*CI*
Migration status and the caregiver												
Non-migrant parents	–	–					–	–				
Father away internationally, mother caregiver	1.12	[0.62,2.03]					2.00*	[1.11,3.60]				
Mother away internationally, father caregiver	0.97	[0.22,4.17]					1.90	[0.48,7.43]				
Both parents away internationally, other caregiver	1.18	[0.52,2.64]					3.53**	[1.43,8.70]				
Stability of the care arrangement												
Non-migrant parents: changed never			–	–					–	–		
Non-migrant parents: changed caregiver ≥1			0.96	[0.52,1.75]					1.76	[0.97,3.17]		
Parent(s) away: never changed caregiver			1.26	[0.66,2.42]					2.47**	[1.31,4.64]		
Parent(s) away: changed caregiver ≥1			0.94	[0.48,1.86]					2.43*	[1.12,5.25]		
Remittances												
Non-migrant parents: no remittances					–	–					–	–
Parents away internationally: yes remittances					0.97	[0.50,1.89]					2.10*	[1.10,4.03]
Parents away internationally: no remittances					0.73	[0.16,3.31]					1.74	[0.32,9.22]
Child age (years)	0.96	[0.83,1.11]	0.95	[0.82,1.11]	0.94	[0.80,1.11]	0.90	[0.77,1.04]	0.90	[0.77,1.04]	0.91	[0.77,1.06]
Child’s happiness status	1.30	[0.99,1.70]	1.32*	[1.00,1.74]	1.20	[0.91,1.60]	1.25	[0.99,1.58]	1.31*	[1.03,1.66]	1.22	[0.96,1.54]
Mother’s education secondary or more	1.18	[0.69,2.01]	1.13	[0.66,1.94]	1.21	[0.69,2.12]	0.95	[0.57,1.58]	0.94	[0.56,1.58]	0.89	[0.53,1.49]
Father’s education secondary or more	0.60	[0.32,1.16]	0.53	[0.27,1.01]	0.44*	[0.22,0.84]	1.03	[0.57,1.86]	0.96	[0.52,1.76]	0.99	[0.55,1.77]
Nr. of siblings living with the child	0.96	[0.87,1.07]	0.97	[0.87,1.08]	0.99	[0.88,1.10]	0.97	[0.87,1.09]	0.97	[0.87,1.08]	0.99	[0.88,1.12]
The child is living with younger siblings	1.37	[0.85,2.21]	1.42	[0.88,2.29]	1.13	[0.68,1.89]	1.31	[0.83,2.08]	1.29	[0.80,2.07]	1.14	[0.70,1.86]
Living conditions are better when compared to other children	1.22	[0.77,1.94]	1.23	[0.77,1.95]	1.14	[0.70,1.85]	1.25	[0.78,2.00]	1.39	[0.86,2.25]	1.22	[0.75,1.97]
Number of people per rooms in the house	0.97	[0.87,1.08]	0.97	[0.87,1.08]	0.94	[0.84,1.06]	1.00	[0.91,1.11]	1.00	[0.90,1.10]	0.98	[0.88,1.08]
Distant relationship with the caregiver	0.88	[0.48,1.59]	0.98	[0.54,1.75]	1.02	[0.54,1.93]	1.37	[0.82,2.28]	1.46	[0.87,2.45]	1.60	[0.95,2.69]
Low–quality schools	2.04*	[1.17,3.55]	1.88*	[1.08,3.28]	1.57	[0.89,2.75]	1.42	[0.83,2.44]	1.36	[0.78,2.36]	1.35	[0.77,2.36]
Private schools	1.12	[0.66,1.91]	1.05	[0.62,1.78]	1.20	[0.69,2.08]	0.61	[0.36,1.03]	0.56*	[0.33,0.95]	0.63	[0.36,1.10]
*N*	696		649		635		715		667		652	
*X* ^2^	20.53		22.47		16.10		23.89		26.20		16.39	
Log-likelihood	−244.53		−241.42		−217.67		−257.54		−250.77		−239.45	

95 % confidence intervals (CI) in brackets; *OR* odds ratios

* *p* < 0.05, ** *p* < 0.01, *** *p* < 0.001

**Table 6:** Children’s perceptions towards *maternal* migration in relation to different transnational family characteristics

	Male						Female					
	Model 10a		Model 11a		Model 12a		Model 10b		Model 11b		Model 12b	
	*OR*	*CI*	*OR*	*CI*	*OR*	*CI*	*OR*	*CI*	*OR*	*CI*	*OR*	*CI*
Migration status and the caregiver												
Non-migrant parents	–	–					–	–				
Father away internationally, mother caregiver	1.01	[0.52,1.96]					1.34	[0.67,2.64]				
Mother away internationally, father caregiver	3.04	[0.73,12.5]					6.84**	[1.60,9.14]				
Both parents away internationally, other caregiver	1.79	[0.78,4.11]					2.98*	[1.18,7.51]				
Stability of the care arrangement												
Non-migrant parents: changed never			–	–					–	–		
Non-migrant parents: changed caregiver ≥1			0.91	[0.46,1.77]					1.78	[0.93,3.38]		
Parent(s) away: never changed caregiver			1.38	[0.70,2.75]					1.76	[0.86,3.56]		
Parent(s) away: changed caregiver ≥1			1.19	[0.58,2.44]					2.94**	[1.30,6.68]		
Remittances												
Non-migrant parents: no remittances					–	–					–	–
Parents away internationally: yes remittances					1.40	[0.70,2.82]					2.31*	[1.14,4.68]
Parents away internationally: no remittances					0.60	[0.11,3.23]					2.18	[0.37,12.6]
Child age (years)	0.96	[0.82,1.12]	0.93	[0.79,1.09]	0.90	[0.76,1.07]	0.91	[0.77,1.07]	0.93	[0.78,1.10]	0.95	[0.80,1.13]
Child’s happiness status	1.53**	[1.11,2.11]	1.44*	[1.05,1.97]	1.46*	[1.05,2.04]	1.41*	[1.06,1.87]	1.46**	[1.10,1.93]	1.40*	[1.05,1.86]
Mother’s education secondary or more	1.19	[0.66,2.17]	1.23	[0.68,2.23]	1.50	[0.79,2.84]	0.94	[0.53,1.65]	0.94	[0.53,1.65]	0.92	[0.52,1.64]
Father’s education secondary or more	0.58	[0.29,1.17]	0.58	[0.28,1.16]	0.39*	[0.19,0.80]	0.70	[0.37,1.34]	0.70	[0.36,1.34]	0.66	[0.35,1.24]
Nr. of siblings living with the child	0.93	[0.83,1.04]	0.92	[0.82,1.03]	0.93	[0.83,1.06]	0.98	[0.87,1.11]	0.97	[0.86,1.09]	0.98	[0.86,1.11]
The child is living with younger siblings	1.47	[0.87,2.48]	1.39	[0.83,2.33]	1.25	[0.71,2.19]	1.21	[0.73,2.03]	1.23	[0.73,2.07]	1.28	[0.74,2.20]
Living conditions are better when compared to other children	1.29	[0.77,2.16]	1.22	[0.74,2.02]	1.16	[0.68,1.97]	1.15	[0.68,1.93]	1.28	[0.75,2.19]	1.26	[0.74,2.17]
Number of people per rooms in the house	1.00	[0.90,1.12]	0.99	[0.89,1.11]	0.98	[0.87,1.11]	0.94	[0.82,1.07]	0.96	[0.84,1.10]	0.93	[0.80,1.07]
Distant relationship with the caregiver	0.92	[0.47,1.79]	0.91	[0.47,1.74]	1.14	[0.56,2.30]	1.26	[0.70,2.24]	1.32	[0.74,2.34]	1.81	[1.02,3.19]
Low–quality schools	1.82*	[1.01,3.28]	1.69	[0.94,3.04]	1.26	[0.68,2.31]	1.06	[0.58,1.95]	0.98	[0.53,1.82]	1.14	[0.61,2.13]
Private schools	1.26	[0.70,2.24]	1.12	[0.63,1.97]	1.06	[0.58,1.93]	0.76	[0.43,1.35]	0.74	[0.42,1.33]	0.81	[0.44,1.48]
*N*	696		649		635		715		667		652	
*X* ^2^	26.24		21.02		19.58		21.75		19.66		22.13	
Log-likelihood	−213.79		−215.86		−191.45		−219.94		−215.73		−203.43	

95 % confidence intervals (CI) in brackets; *OR* odds ratios

* *p* < 0.05, ** *p* < 0.01, *** *p* < 0.001

Multilevel models were considered but not fitted because they failed the test for intraclass correlation (ICC) at the school and city levels. Nevertheless, indicators that measured the type and the quality of schools were included in the analysis to account for the unobserved characteristics at the school level. Indicators in all models were tested for multicollinearity and none was found; the variation inflation factors (VIF) (1–1.5) and the tolerance values (0.8–0.9) were average.

## Results

### Descriptive Statistics

Briefly, we only present the descriptive results pertaining to the dependent variable and the transnational characteristics. Table 1 provides a descriptive overview of all indicators included in the analysis. Within the sampled population, more children reported positive perceptions towards paternal migration compared with maternal migration (36.84 and 27.16 %, respectively). Overall, 23.01 % of children had one or both parents abroad and in a stable marital relationship. In addition, 9.13 % of all children were living in divorced or separated families in which at least one parent was abroad. Of the children with a migrant parent in a stable marital relationship, 14.53 % had their father abroad and were cared for by their mother, 2.76 % had their mother abroad and were cared for by their father and 8.86 % had both parents abroad and were cared for by a non-parental caregiver. Given the small number of children who are cared for by a non-parental caregiver, we are unable to further split this sample according to who these caregivers are. However, our data reveal that when both parents migrate, children are usually cared for by an uncle/aunt, a grandparent or other siblings. Data further show that 28.49 % (12.46 + 16.03 %) of children in migrant and non-migrant families had changed their caregiver at least once. Most children in transnational care received remittances; only 2.87 % of them reported the absence of a monetary flow from their parents abroad.

Differences in perceptions could be observed among male and female children in the characteristics of their transnational lives, as shown in Table 2. Overall, children perceived paternal migration more favourably than maternal migration. Positive perceptions towards paternal migration did not differ across boys and girls by the marital status of the migrant parents. However, parental divorce considerably reduced the positive views towards maternal migration, especially among girls. Among boys, the perceptions towards parental migration were highest when both parents were away, but girls held this view only with regard to paternal migration. Children in non-migrant families who changed caregivers at least once were less inclined to favourably perceive parental migration, especially that of mothers. When the change in caregivers occurred among children in transnational care, the positive perception towards parental migration increased, especially in favour of paternal migration. The absence of remittances resulted in more positive perceptions towards parental migration, especially in favour of paternal migration. Notably, girls in non-migrant families had consistently more negative perceptions towards parental migration, especially with regard to maternal migration.

### Regression Results

Tables 3 and 4 summarize the stepwise regression results for the relationship between children’s perceptions towards paternal and maternal migration based on the marital status of migrant parents residing abroad. This analysis controls in a hierarchical manner for child characteristics (Table 3, Models 1a & 1b; Table 4, Models 4a & 4b), family factors (Table 3, Models 2a & 2b; Table 4, Models 5a & 5b) and school type indicators (Table 3, Models 3a & 3b; Table 4, Models 6a & 6b). The results demonstrate that the relationship between the type of separation and children’s views of parental migration differed for male versus female children. For female children, having a migrant parent in a stable marital relationship corresponded to higher odds of perceiving maternal and paternal migration more positively. Notably, the odds were higher and significantly stronger when girls assessed paternal migration compared with when their mothers had migrated. These results partially support Hypothesis 1a. At the same time, parental migration and problematic marital status did not correspond to significant differences on how male and female children in transnational care perceived maternal and paternal migration compared with the perceptions of children in non-migrant families. Based on this evidence, we reject Hypothesis 1b.

The second analysis employs three distinct characteristics pertaining to children’s transnational care arrangements (Tables 5 and 6). As in the first analysis, the results are presented for male and female children separately. The first characteristic looks at the parental migration status and the caregiver : non-migrant parents, a father abroad and a mother caregiver, a mother abroad and a father caregiver and both parents abroad and a non-parent caregiver (Table 5, Models 7a & 7b; Table 6, Models 10a & 10b). The results show that parental migration and the caregiver had specific bearing on how female and male children perceived paternal and maternal migration. Female children were more likely to perceive paternal migration as good/very good when the father was away and the mother was the caregiver. Hypothesis 2b is therefore confirmed in this particular context. Contrary to Hypothesis 2a, the migration of a mother, when the father was the caregiver, also corresponded to a higher probability that girls would perceive maternal migration as good/very good. In other words, female children were more likely to have positive perceptions of migration towards the parent who was abroad when the other parent was the caregiver in Ghana. Moreover, contrary to Hypothesis 2c, female children with both parents abroad were also more likely to have a positive view towards parental migration, especially with regard to paternal migration. For male children, no form of parental migration and the caregiver status appeared to be significant.

The second characteristic focused on the stability of the care arrangement and consisted of children in non-migrant families who had never changed caregivers, children in non-migrant families who had changed caregivers at least once, children with parents abroad who had never changed caregivers and children with parents abroad who had changed caregivers at least once (Table 5, Models 8a & 8b; Table 6, Models 11a & 11b). A significant link exists between the frequency of change of the care arrangement and more positive perceptions of paternal and maternal migration but only among the female children in transnational care. Female children with migrant parents who had never changed caregivers were also found to have more positive perceptions towards fatherly migration. We therefore reject Hypothesis 3.

The third characteristic assessed the availability of remittances for children in transnational care (Table 5, Models 9a & 9b; Table 6, Models 12a & 12b). The results show that female children who reported receiving remittances from parents abroad were more likely to perceive both maternal and paternal migration as good/very good, but this finding was not replicated for male children. Additionally, the absence of remittances was not associated with more negative perceptions towards parental migration among children in transnational care. Therefore, Hypothesis 4 is only partially supported.

## Discussion

Drawing on transnational family studies and on sociological reflections that consider children as autonomous social actors in the realm of migration, we present three reflections that aim at emphasizing the contribution of this study to the literature.

First, complex forms of life of children in transnational care matters when assessing views towards parental migration. Specifically, we found that parental migration in the context of marital stability positively influenced the perceptions of female children towards maternal and paternal migration. Interestingly, however, the context of marital dissolution did not bear negative consequences on how children perceived parental migration, as the literature suggests. Previous research shows that children experience parental migration and parental divorce in different ways and that parental divorce has a worse impact (Nobles 2011). In the context of this study, it could mean that children are able to dissociate parental migration from parental absence following divorce, except perhaps for when divorce is the direct result of the migration process. Because of sensitivity issues, our data did not account for details of parental divorce, such as whether the divorce occurred before or after the migration event. A recent study looked at the prevalence of divorce among Ghanaian migrants in Europe and found that it was not more prevalent among couples who migrated than among those who stayed in Ghana (Caarls and Mazzucato 2015). This evidence may imply that divorce and migration represent random occurrences among Ghanaian couples and that children may perceive the two events as uncorrelated experiences and are able to dissociate their feelings for migration from those regarding marital discord.

When parents migrate but are in a stable marital relationship, different characteristics linked to children’s transnational care may influence how children perceive parental migration. Specifically, we found that female children perceived the migration of the parent more positively when the assessed parent was abroad while the other parent remained as the caregiver. In addition, girls were found to perceive maternal and paternal migration more positively when both parents were abroad. Ghanaian male children in transnational care did not have differing perceptions towards parental migration from those of children in non-migrant families. One implication of these findings is that they counter the existing view in the literature that associates maternal migration with more distress among children (Parreñas 2005). Although previous studies emphasize the negative effects of maternal migration, most of the evidence came from research that was primarily conducted with adults, leaving children’s perspectives largely unobserved. However, as recent research demonstrates, children’s perspectives may differ significantly from those of adults (Jordan and Graham 2012). Our findings are in line with those from other recent studies that used children’s reports and found that Ghanaian children were not emotionally affected when their mothers or both parents migrated (Mazzucato and Cebotari 2016; Mazzucato et al. 2015a). Another implication of the obtained results is that they emphasize the role of the caregiver for children in transnational care. In this study, we look not only at who is the migrant parent but also at the caregiver of children in transnational care. In the analysed sample, most children of migrants lived with a parent or a close family relative. When parents migrate and children stay in the care of a family member, children experience the benefits of living in a familiar context in the home country. Even when children had experienced a change in their care arrangements, we still did not find negative consequences for how children perceive the migration of their parents. To a certain extent, this finding may imply that a family environment provides security for children in transnational care and that changing caregivers would have little impact on how children perceive migration as long as these changes occurred within a familiar family setting in Ghana.

Second, our findings show gender-specific outcomes that warrant further exploration. Specifically, the results are consistent in showing that female children perceived maternal and paternal migration more positively than did male children. This evidence is intriguing but perhaps not entirely surprising. Studies show that parental migration is beneficial for female children because migrant parents often entrust them with increased decision making with regard to the care of younger siblings, the use of remittances and the overall household expenditures (Parreñas 2005). Many of the Akan communities in Ghana, from where a significant share of migrants originate, are traditionally matrilineal; women enjoy a high degree of independence, including keeping separate budgets and managing their own economic affairs. These cultural traits may trickle down to the daughters of female migrants, who may be empowered by local values and attitudes to control their own lives, including potential aspirations to follow their parents into migration.

The finding that male children do not show similar views to those of female children also requires examination. Studies show that boys often struggle to find appropriate means of emotional expression with regard to parental migration (Vanore 2015). Specifically, male children have a tendency to avoid expressing themselves about emotional matters because of the thought that their views would make them seem weak. We measured children’s views towards parental migration using questions that captured internalized perceptions, and therefore, the boys’ responses might not have easily reflect on their feelings. Future research would benefit from additionally exploring the distinctions between female and male children with regard to attitudes towards parental migration.

Third, this is the first empirical analysis of how children perceive parental migration in an African context. In itself, we contextualize the results against the local family norms in Ghana. Most of the existing evidence comes from studies conducted in Latin American and Asian countries where the nuclear family model is strong. These studies describe the stigma associated with parental migration, which leads to feelings of abandonment and unhappiness among children in transnational care (Asis 2006; Dreby 2007; Jordan and Graham 2012; Parreñas 2005). In Ghana, as in most Sub-Saharan African countries, the practices of child fostering and social parenthood are widespread, making it common for children to live in the care of someone other than their biological parents. This means that in Ghana, children may not feel stigmatized in their social environment when they live separated from their parents because of migration (Poeze and Mazzucato 2014). The prevalent practice of child fostering also means that for some children, parental migration may not involve a change in caregiver if the child was being fostered prior to parental departure. We therefore postulate that the absence of negative attitudes towards migration is due to the widespread norms of child fostering and social parenthood that help children develop resilience in the aftermath following parental migration.

This study is not without limitations. Children’s perceptions are measured with static indicators and we are unable to further explore child feelings given the quantitative nature of our study. Furthermore, we were unable to collect reliable data on important factors such as the length of the child’s separation from the migrant parent. Because the data were collected by asking children themselves, many of them, especially the younger children, had difficulty remembering the dates when their parents had migrated. Previous studies suggest that children feel most affected by migration immediately following parental departure (Vanore 2015). Future studies must explore this covariate. Another limitation refers to the highly selective process of migration; those who migrate do not represent a random proportion of the population in the country. Instead, migrants have specific characteristics that make them more prone to migration. A number of observable characteristics that may influence the choice to migrate, including the parents’ education and their living conditions, were included in the analysis, but other factors such as the socio-economic status prior to parental departure could not be included given the cross-sectional nature of the data. Therefore, the findings show correlations but not causation, and we caution readers to keep this in mind when reading this article. A final limitation relates to our sampling procedure. The data were collected in schools, and as a result, we did not account for children in transnational care who dropped out of school.

Despite these limitations, this study presents important evidence on how children experience parental migration in an African transnational context. Specifically, we show that male and female children tend to perceive parental migration differently but not in a negative way. The different characteristics of children’s transnational lives nuance the results and demonstrate that child agency in the context of migration is not unidimensional and must be approached in its complexity. Insights from this study can be used to inform a policy dialogue on how transnational families function across borders.
